# Social Representations of pregnancy among trans men

**DOI:** 10.1590/0034-7167-2024-0094

**Published:** 2025-01-10

**Authors:** Danilo Martins Roque Pereira, Ednaldo Cavalcante de Araújo, Sheyla Costa de Oliveira, Anderson Reis de Sousa, Mariana Mercês Mesquita Espíndola, Milka Gabrielle de Lira Nóbrega West, Marclineide Nóbrega de Andrade Ramalho, Joecio Cordeiro Cardoso

**Affiliations:** I Universidade Federal de Pernambuco. Recife, Pernambuco, Brazil; II Universidade Federal da Bahia. Salvador, Bahia, Brazil; III Centro Universitário FMABC. Santo André, São Paulo, Brazil

**Keywords:** Social Theory, Sexual and Gender Minorities, Pregnancy, Nursing Care, Health Education, Teoría Social, Minorías Sexuales y de Género, Embarazo, Atención de Enfermería, Educación en Salud

## Abstract

**Objective:**

to understand the meanings and experiences of pregnancy among trans men in light of the Theory of Social Representations.

**Methods:**

this is a qualitative, descriptive and exploratory study, carried out with trans men selected for convenience and availability. Data production took place from September to October 2021, via the Google Meet^®^ platform, based on interviews with a semi-structured script. Interview lexicographic textual analysis was performed using the Reinert method and instrumented by IRAMUTEQ version 7.0.

**Results:**

social representations of pregnancy involved a wide field of meanings, in which efforts were articulated to accept opportune and solitary pregnancy, fear of the parturition process and influence of physical and emotional changes.

**Final considerations:**

the study reinforces the importance of advanced nursing practice in assisting the pregnancy-puerperal cycle, based on the perspective of comprehensive care, equity in access to services and respect for differences.

## RESUMEN

**Objetivo:**

comprender los significados y vivencias del embarazo entre hombres trans a la luz de la Teoría de las Representaciones Sociales.

**Métodos:**

se trata de un estudio cualitativo, descriptivo y exploratorio, realizado con hombres trans seleccionados por conveniencia y disponibilidad. La producción de datos se realizó de septiembre a octubre de 2021, a través de la plataforma Google Meet^®^, a partir de entrevistas con un guión semiestructurado. El análisis textual lexicográfico de las entrevistas se realizó mediante el método Reinert e instrumentado por IRAMUTEQ, versión 7.0.

**Resultados:**

las representaciones sociales del embarazo involucraron un amplio campo de significados, en los que se articularon esfuerzos por aceptar el embarazo oportuno y solitario, el miedo al proceso del parto y la influencia de los cambios físicos y emocionales.

**Consideraciones finales:**

el estudio refuerza la importancia de la práctica avanzada de enfermería en la asistencia al ciclo embarazo-puerperal, desde la perspectiva de la atención integral, la equidad en el acceso a los servicios y el respeto a las diferencias.

## INTRODUCTION

In Western society, paternal pregnancy is generally seen as an “unlikely” possibility for “trans men”, as reproduction is considered impossible for individuals conceived through the idea of abjection. This results in a condition called “symbolic sterility”, characterized by the denial of the right to choose reproduction and to exercise parenthood, whether biological or not^(1)^.

In this scenario, trans men who have gone through pregnancy or are in the gestational process give meaning to the events of pregnancy, childbirth and breastfeeding as intrinsic elements in the construction of their masculinity. This occurs even though individuals have a vagina, uterus and ovaries, thus defying cisheteronormative reproductive norms^(2,3)^.

Research has shown that trans men who have experienced pregnancy present specific demands related to the effects of hormone use, surgical interventions and changes in body perceptions that occur during this period^(4)^. Allied to this, transphobia has promoted an “expulsion” of this public from healthcare services, based on non-respect for social names, inappropriate use of treatment pronouns, inappropriate questions, lack of information that takes into account their health specificities, among others, also creating barriers to access to comprehensive health^(5,6)^.

In the context of care, from the perspective of health promotion, health education emerges as a resource to be used by nurses in the role of health educators, with the ability to enhance pregnant trans men’s reflection about their representations arising from their experiences, providing criticality and the ability to understand weaknesses and abilities to overcome the difficulties that may present them at this stage of life^(7,8)^.

In this regard, investigating the social representations of trans men who have become pregnant or are going through pregnancy enables an understanding of their interpretations and information about this phenomenon from the perspective of common sense. This, in turn, allows nurses to develop health practices and engage in the fight against institutional transphobia^(8)^. It is understood that the Theory of Social Representations (TSR) offers a theoretical framework to understand the daily life of specific groups, enabling the creation of practical knowledge by being integrated with experiences, which include historical, cultural and spatial context, and by guiding individuals’ communications and behaviors^(9)^.

Therefore, given the above, this study had the guiding question: what are the meanings and experiences of pregnancy among trans men?

## OBJECTIVE

To understand the meanings and experiences of pregnancy among trans men in light of the TSR.

## METHODS

### Ethical aspects

The research project was submitted to the *Universidade Federal de Pernambuco* (UFPE) Health Sciences Center (HSC) Research Ethics Committee (REC), under Opinion 4,862,503, approved in 2021, in compliance with Resolution 466/12 of the Brazilian National Health Council (CNS - *Conselho Nacional de Saúde*), which regulates guidelines and standards that deal with research with human beings. After approval of the research project at REC/UFPE, empirical data production began. Participant identification was anonymized using self-declared pseudonyms at the time of information collection.

### Study design

This is a qualitative, descriptive and exploratory study, structured according COnsolidated criteria for REporting Qualitative research (COREQ) guidelines and anchored by TSR of Serge Moscovici and followers. In order to access knowledge about the reality of a given social group, the TSR allows nursing to guide care and conduct healthcare based on the recognition of its characteristics and specificities^(10)^.

### Study setting

The research was conducted at a national level, involving individuals linked to prominent institutions for the Lesbian, Gay, Bisexual, Transvestite and Transgender (LGBT) population in Brazil. These institutions include the Brazilian National LGBTI+ Alliance, the *Ambulatório LGBT Patrícia Gomes*, in Recife, Pernambuco, and the LGBT Health Coordination of the State of Pernambuco. The selection of these research sites followed intentional sampling criteria, aiming to incorporate essential characteristics to develop the research and cover the population of interest.

### Data source

Study participants were trans men who met the following criteria: 1) inclusion: trans men who were pregnant or who were pregnant and aged 18 years or over; 2) exclusion: pregnant trans men or those who have gone through pregnancy, who had total visual and/or hearing impairment, given the researcher’s inability to understand Libras (Brazilian Sign Language) and Braille, and with health problems that hinder understanding and participation in the study or who did not have technological resources to carry out the research (computer, cell phone, tablet, internet, among others), considering the pandemic context. Participants were selected based on convenience and availability to be interviewed. The choice of this technique was due to the need to gather the population of interest, as they have peculiar characteristics or are difficult to identify^(11)^. Seven participants were included through progressive insertion, based on theoretical saturation of responses to semi-structured interviews^(12)^.

### Data collection and organization

To collect information, which took place from September to October 2021, the semi-structured individual interview technique was used via telephone call or Google Meet^®^. A script helped conduct the interview and gathered questions about aspects relevant to the study objective. It was composed of the guiding questions as follows: 1. Tell me about how your pregnancy was. 2. Tell me about the changes (bodily, social, emotional, psychological, among others) from the beginning to the end of pregnancy. 3. Tell me about your healthcare provided by the health team for reception and care during pregnancy. 4. Do you know other trans men who have become pregnant or are pregnant?

Subsequently, meetings were scheduled with the target audience via telephone call or messaging applications, such as WhatsApp^®^, Instagram^®^, Facebook^®^ or email, based on research participants’ availability. It was recommended that participants, at the time of the interview, remain in a private and silent environment, criteria necessary for there to be a minimum of external interference. Respondents were informed about the research objective through the Informed Consent Form (ICF) and about the need to sign the Image and Testimonial Release and Consent Form, made available by Google Forms^®^. Moreover, a form was used to fill out participant characterization data, made available before the interviews and on the main social networks (Facebook^®^, Instagram^®^ and WhatsApp^®^). Individual online interviews were consented and recorded on video for a maximum of two hours, using the resource provided by Google Meet^®^. After each transcription, interviewees were sent for content validity, giving them the opportunity to add or remove information, if necessary. It was requested that cameras were turned on at the time of online interviews so that discursive pauses and intonations could be identified by the researcher as well as facial expressions, gestures and other body language.

### Data analysis

Empirical information produced by individual interviews was subjected to text analysis using the Reinert method of text segment classification (TS), instrumented by the free software *Interface de R pour les Analyzes Multidimensionnelles de Textes et de Questionnaires* (IRAMUTEQ) version 7.0. Descending Hierarchical Classification (DHC) was used as a technique for processing information^(13)^.

## RESULTS

The universe of meanings of seven trans men’s social representations about pregnancy is described below, supporting the questions proposed in this research, translating the “common sense” of this group. For greater data richness, Chart 1 presents characterization according to the profile of trans men’s pregnancy-childbirth cycle, in order to visualize their profile and better situate the sample, since the object of any social representation is defined based on the phenomenon and the chosen group^(14)^. These were identified by self-declared pseudonyms at the time of the interviews.

**Chart 1:** Characterization of the profile of the pregnancy-childbirth cycle of trans men participating in this study. Recife, PE, Brazil, 2022

	How became pregnant	Used masculinizing hormone during pregnancy	Pregnancy was planned	Attempts to terminate pregnancy	Type of childbirth	Goes/went to prenatal appointments	If “yes” to the previous question, which professional provided prenatal care?	Number of children
**I1**	Penis-vagina sexual intercourse	No	No	No	Vaginal	Yes	Doctor	1
**I2**	Penis-vagina sexual intercourse	Yes	No	No	Cesarean section	Yes	Doctor	1
**I3**	Penis-vagina sexual intercourse	No	No	No	Cesarean section	Yes	Doctor	1
**I4**	Penis-vagina sexual intercourse	No	No	No	Cesarean section	Yes	Doctor	1 biological and 2 non-biological
**I5**	Penis-vagina sexual intercourse	No	Yes	No. Reports previous missed miscarriage.	Cesarean section	Yes	Doctor	1
**I6**	Penis-vagina sexual intercourse	No	No	No. Reports previous miscarriage.	Vaginal delivery is intended.	Yes	Nurse	0
**I7**	Penis-vagina sexual intercourse	No	No	No	Vaginal	Yes	Doctor	1

The analysis *corpus* of this study was composed of seven texts, submitted to analysis to obtain DHC, divided into 1,172 TS, relating 3,883 words, with 41,094 occurrences. DHC obtained 92.75% of the total TS, corresponding to 1,087 TS, generating four classes. Subsequently, classes are described following the order of partition and proportion they represent in relation to the total *corpus*.

Class 1 (Chart 2), “Discovery of pregnancy and its implications”, corresponds to 41.12% of TS, relating to the moment when trans men discover pregnancy and the consequences of this event in their lives. The meaning of this class concerns a moment in which the signs of pregnancy are confused with various comorbidities, attributing the confirmation of an unexpected pregnancy to the feeling of “falling apart”, “insecurity” and “not knowing what to do”, in addition to abortion as an alternative to termination. I4’s report stands out, which talks about the occurrence of a “corrective rape” and which resulted in pregnancy, this being the only report that highlights this fact, therefore, essential in reflections on implications for care.

**Chart 2:** Class 1: Discovery of pregnancy and its implications. Recife, PE, Brazil, 2022

Identification	Statement
**I1 - Bernardo**	[...] *when the news came, it was like the feeling, I don’t know, as if I was inside a house and it had just collapsed* [...] *because the first thing I thought was, “What am I going to do?”. And the other parent’s first reaction was, “Ah, let’s take it off!” He didn’t come and say, “So? Will you want it?”* [...] *and I blamed myself for not having been more responsible, and then comes the fact that I had just come out of depression, a bad relationship, and I was afraid of losing my job, because I had just entered the company* [...].
**I2 - Iago**	[...] *the doctor looked at my face and said, “Can I ask you a question? Is there a possibility that you are pregnant?”. I said, “Look, I have sex without a condom, but I take hormones and they’ve been telling me for years that I can’t have children because I’m sterile”. I said, “Look, I went to the hospital in my city; he told me that I had very strong bacteria in my stomach and even gave me the medication saying that this is gas”. She said, “That’s not gas, no, if that’s not a child, I’ll stop being a doctor!”. When she said that, my face fell to the floor* [...].
**I3 - Coutinho**	[...] *before pregnancy, I had been without hormones for two months, I had been using Deposteron for two years. It wasn’t planned, but it was desired. My fiancée was not using hormones or blockers either.* [...] *I found out a week before my mastectomy... and it wasn’t a happy moment* [...] *it was a moment when I felt like I no longer owned my body, you know? ... I wasn’t prepared for a pregnancy. At that moment, I was preparing to fulfill another dream which was a mastectomy... my mental health went to zero* [...].
**I4 - Lessa**	[...] *about pregnancy, I never imagined being a father or a mother, right? At the time, it was still read as mother, but I had never imagined that possibility, and things happened in a way I didn’t expect. I suffered what we now know as corrective rape... and, during the violence, the attacker said he was going to show me what it was like to be a real man and at times he said he was going to make me be a real woman* [...].
**I5 - Brandon**	[...] *our child’s pregnancy was extremely planned* [...] *planning occurred in a very autonomous way, okay?* [...] *my partner and I decided at some point that we would go through this process, and then we started researching* [...] *and we saw that we would need to stop taking hormones in the process and we stopped* [...].
**I6 - Matheus**	[...] *the only point that remained in my head, whether I would be able to conceive or not, had nothing to do with myself, me and my body. It was more of a relationship between me and society, how I was going to deal with social response* [...] *if I was going to be able to deal with it* [...].
**I7 - Azevedo**	[...] *pregnancy wasn’t something planned, it was really scary. At first, it was a bit of a shock for me. At first, there were thoughts of taking it off* [...] *it was during a season when I had to take a break from my hormones and it ended up happening* [...] *when I discovered that I was pregnant* [...] *I was already expecting my child, I was already three months old and I had no idea* [...] *at the beginning, I was a little scared, mainly because of the fear of things that I would pass, prejudice* [...].

Class 2 (Chart 3), “Family relationships, loneliness and lack of support”, corresponds to 27.97% of TS, relating to how trans men established their family ties during pregnancy, reflections on support from partners, questions about pregnancy about “whose child it would be”, attribution to the experience of pregnancy as a moment of “deprivation” and “solidarity”.

**Chart 3:** Class 2: Family relationships, loneliness and lack of support. Recife, PE, Brazil, 2022

Identification	Statement
**I1 - Bernardo**	[...] *they were extremely happy* (with the pregnancy), *and, at the time, many still didn’t see me as Bernardo, and called me by my old name, so they were happy because I was another woman in the family having a child* [...] *but always with this vision that now that you’re going to have a child, maybe you’ll go back on the transition, which was a hope that people had, you know? To this day, there are people who* [...].
**I2 - Iago**	[...] *I was very angry with my daughter’s father and ended up going back to the hospital* [...] *I said, “Do you want to do the DNA test, we’ll do it when the child is born* [...] *if you want to do it now, you can do it. From the fourth month of pregnancy, you can take a DNA test!* [...] *I want it because you are doubting my word. I have no reason to say that she was your daughter”* [...].
**I3 - Coutinho**	[...] *in my head, I wasn’t capable of being a home for a baby, because I wasn’t even understanding myself as a human being, you know?* [...] *I couldn’t look at myself and think that this was being beautiful, you know?* [...] *I just thought that I wanted it to go away soon, you know?* [...] *it’s all lonely and we spend a lot of time with ourselves* [...].
**I5 - Brandon**	[...] *when they* (the family) *found out, they were very happy. I even told my mother that I was going through pregnancy* [...] *but then came demands like “you’re going to shave your beard, right?”, like, since you’re going through pregnancy, then you’re going to detransition* [...] *I explained that it was not and that, in fact, it was transitioning that showed me pregnancy as a possible process. There was a lot of lack of knowledge in this process* [...] *of not understanding pregnancy as the possibility of my masculinity* [...].
**I6 - Matheus**	[...] *and so I’m a little distant from my family on my mother’s side, but when I told them, there were no questions. Nobody asked anything at all, I’m happy. My father* [...] *I went to tell him, “Oh, maybe there’s another new member of the family coming and you’re going to be a grandpa again”. Then he said, “What do you mean? Who is the parent, who is the parent?”, so I said, “What do you mean, who is it, bro? You are crazy? It’s mine!”* (laughter). *And then he said, “But really? I didn’t think you guys had sex like that”* [...] *that blew his mind* [...].
**I7 - Azevedo**	[...] *the violence I experienced during pregnancy affected me a lot, because I went out on the street just because I needed to work. Other than that, because I was so afraid of these things, of violence again or of the prejudices that I was already suffering, of people seeing my belly and not understanding* [...] *I deprived myself a lot, I was locked inside the house the entire pregnancy until after I had my child* [...].

Class 3 (Chart 4), “Senses and experiences during healthcare”, corresponds to 19.96% of TS and concerns the meanings built on experiences during healthcare, difficulties during reproductive planning in a trans-centered relationship, discomfort in sharing “feminine” spaces during prenatal care, denial of rights to pregnant men, reproduction of transphobic speeches by healthcare professionals and prenatal care between the public and private sectors. It is also evident that pregnancy is an experience of “self-knowledge”, “positive and negative changes” and “pedagogical”, based on the recognition of the potential of their body and the understanding of pregnancy as a phenomenon that constitutes their masculinity. Fear of childbirth becomes present and is related to the body’s ability to have a vaginal delivery, but, mainly, to the occurrence of obstetric and transphobic violence in healthcare institutions, given that care for the pregnancy-puerperal cycle takes place in places designed for the female and cisgender public, and, in the case of transmasculine pregnancies, this aspect can worsen.

**Chart 4:** Class 3: Senses and experiences during healthcare. Recife, PE, Brazil, 2022

Identification	Statement
**I1 - Bernardo**	[...] *as I was working, and at work I had health insurance, I said, “I’m going to take the easiest path theoretically”* [...] *they put a lot of terror into the issue of childbirth through the SUS, you know? From treatment, and how it’s done and everything, and that’s with cis women, imagine a trans man* [...] *and then I was very scared of what it would be like, you know?* [...] *for me, one of the biggest defects of healthcare personnel is clearly that they don’t know how to take care of a trans body, and then we kind of have to teach them, you know?* [...].
**I2 - Iago**	[...] *it was something that really bothered me, being in a very feminine environment* [...] *I was very scared, really scared, when I arrived, I was shaking* [...] *there was a nursing technician who... woke up in the morning and said, “I’m not going to call you “he”, no, you’re “she”! A man doesn’t get pregnant, you have a pussy”. Then the girls in the room said “Don’t you care about it!”. Then she said, “You’re going to have a child, you’re going to upset the child* [...] *you want to be what you can’t! Look at you. You are a woman* [...] *you have a pussy. You don’t have a dick, no!”* [...] *then my blood pressure went up. I already started to feel bad* [...].
**I5 - Brandon**	[...] *about the care provided by my obstetrician* [...] *what I will face are some institutional barriers... for instance, she only had one women’s bathroom in her office. She only had one bathroom with a female sign. So, these are things that we often draw attention to, which are symbolic micro-violences that tell me that that place doesn’t belong to me,* [...] *she requested that I take a certain vaccine during the pregnancy period and I went to a Family Health Unit and, when I got there, I basically had to go through an interrogation with a community health worker, and he didn’t understand that I was a trans man* [...] a*nd he made it difficult for me to get that vaccine...* [...] *he understood, even though I was repeating it to him, that I was pregnant; he thought I was being a fraud there, right?* [...].
**I6 - Matheus**	[...] *at the family clinic* [...] *it was wonderful there, so the girl never insulted me, she just had doubts, she didn’t understand everything right, and they had to make a change, because, in the SUS, if you are from male, you cannot enter into the protocol in the line of care for pregnant women, right? So, they had to make a change* [...] *they changed my gender to female, but my name remained the same, and then the boy told me that he would send an email to the person responsible for creating “SUS thing”. I highly doubt it, but I thought it was very cute anyway, it’s their care, right? ... now, the nurse, specifically, was terrible, horrible* [...] *he didn’t know if it was me who was pregnant, if it was my partner, he was confused. He wanted to know my registered name in every possible way, and I didn’t say my registered name, he only addresses me in the feminine* [...].
**I7 - Azevedo**	[...] *I already had a completely masculine physique, a beard* [...] *and when my belly started to grow, then it was a much bigger blow. I had difficulty with people giving up seats on the bus and I worked throughout my pregnancy. At the very end, close to having the baby, I got my license. Every day when I took the bus, there were two episodes but I had people who gave me a seat. I was always on my feet and always taking risks* [...].

Class 4 (Chart 5), “Body and emotional changes and strategies for hiding pregnancy”, corresponds to 10.95% of TS, relating to the physical and emotional changes resulting from the gestational process and the influence these have on the experience of these men. This class considers aspects such as increased desire to undergo mastectomy surgery after pregnancy, breast enlargement as one of the greatest discomforts, use of “binder” or “micropore tape” and bra during pregnancy, discomfort with the voice, use of cold coats as a strategy to hide their “pregnant belly”, swelling and pain in the surgical scar from masculinizing mastectomy, not feeling sexually desired by the partner and not recognizing oneself in the image reflected in the mirror.

**Chart 5:** Class 4: Body and emotional changes and strategies for hiding pregnancy. Recife, PE, Brazil, 2022

Identification	Statement
**I1 - Bernardo**	[...] *the fact that I’m pregnant, whether or not my body changes, whether I want it or not* [...] *let’s say, like, it’s the most feminine thing a trans guy can do... I think what screamed the most was the chest* [...] *was the worst for me, because everything else was very smooth, because I’ve always been fat, so gaining weight wasn’t a problem and I deal very well with the fact that I have a vagina* [...] *now the breast, the breast has taken hold* [...] *it was very complicated to deal with and use the breast afterwards to breastfeed my son* [...].
**I2 - Iago**	[...] *the breast swelled with milk, so it looked really ugly. I chose to wear the bra. It bothered me* [...] *but, for my daughter’s sake, I wore a bra and fed her in the corners, and the others kept looking because I had a beard* [...] *one thing that bothers me to this day is my voice.* [...] *the voice gives everything away* [...].
**I3 - Coutinho**	[...] *accepting the changes, the dysphoria, every day I have to understand that it is temporary, that I will go back to being what I was and that all of this is for the greater good, which is my daughter’s health* [...] *since the first appointment, I was instructed to no longer use tape, binder… anything that compresses the breasts. So, that already hurt me a lot. So, I wore a coat and almost nothing was showing yet. I could wear a tight, not tight top* [...] *to disguise it a little with the coat. That was a very painful change* [...].
**I5 - Brandon**	[...] *when I thought about pregnancy, I said, “Look, I’m going to go through this process”* [...] *I had some things that I was willing to do and others that I became willing to do, such as breastfeeding... the binder, I always used it and, during pregnancy, I tried to reduce it a little at the end, because I felt a lot of back pain* [...] *and then the technology I used was the old coat, right? I wore bulkier coats* [...] *it was the way I felt most comfortable going out on the street without often being identified* [...] *it was also a way of protecting my safety, because it was very common when people realized that I was a trans man, you know? I received repressive looks* [...].
**I6 - Matheus**	[...] *I’m dealing with the changes very well, and I’m enjoying seeing my belly grow, I’m happy with it* [...] *and it’s not affecting my masculinity in any way. I’m fine as the man I am. I know the possibilities that my body is capable of and that doesn’t make me any less of a man* [...].
**I7 - Azevedo**	[...] *I’ve always had dysphoria about this, mainly because of my breasts, and it was something that used to bother me a lot and when it became even bigger during pregnancy* [...] *micropore tape, it really hurt me* [...] *I wore it all day, whenever I had to go out for something like that, I only took it off when I got home, when I went to bed and took a shower, that sort of thing.* [...] *and I wore it and felt much better to go out and present myself financially* [...] *I kind of didn’t accept my body changing with that, I didn’t accept that inside me* [...] *then I started to spend even more time with the blouse cold, wearing loose clothes, big clothes to hide as much as possible as I could* [...].

## DISCUSSION

### C1) Discovery of pregnancy and its implications

It is observed that the social representations of the impact of discovering pregnancy among trans men in this study who became pregnant in an unexpected way were anchored in feelings of “insecurity”, “falling apart”, “lack of responsibility” and “not knowing what to do”, crossed by “fear of social response”, in the face of expectations and acceptability regarding a pregnant transmasculine body. It is possible to consider that this category is associated, in some contexts, with the idea culturally constructed and dissipated by Western society about pregnancy as a biological, feminine and cisgender phenomenon. In this case, “cisheteronormative pregnancy” appears as an objectification of this reality and represents a universal and homogeneous character before men and women, considered as the model of acceptability of conceiving a child, especially the ability to give birth as a founding characteristic of femininity supported by social constructions of gender, highlighting socially pre-established roles, such as notions about motherhood and fatherhood.

It is clear that the contents of social representations about the impact of discovering pregnancy among trans men involve a wide field of meanings, in which their efforts to “accept” the opportune pregnancy are articulated through an individual and subjective process of empowerment by “leaving oneself aside”, by opting to continue the gestational process based on the recognition of this phenomenon as a potentiality of their body, taking risks in facing situations of transphobic violence and the challenges intrinsic to the pregnancy cycle.

Corrective rape may appear as a mechanism by which trans men are vulnerable and, consequently, subject to becoming pregnant, which, in this case, is characterized as sexual violence in which abusers aim to “correct” victims’ sexual orientation or gender identity, as presented in one of the cases in this study. It should be noted that rape is a crime, provided for in the Brazilian Penal Code, in its Article 213, as amended by Law 12,015 of 2009^(15)^.

Questions in favor of or against abortion practices are present among healthcare professionals, even when it comes to legal abortion^(16)^. From the perspective of social representations, condemnation of abortion practices is related to moral, social and cultural standards, crossed by religion and legislation^(17)^. However, in this study, abortion appears as a viable alternative for maintaining the *status quo* promoted by the traditional family and the reproductive pillars that are based on cisheteronormativity.

Studies already demonstrate reports of spontaneous abortion and induced abortion among trans men and that it is related to several factors; therefore, it is necessary to recognize the need for healthcare services to guarantee adequate and humanized management of risk situations and comprehensive care. The violence and prejudice suffered in healthcare services by people who choose abortion are not exclusively directed at trans men, but are a reality that violates the right to choose of men and women who become pregnant^(18,19)^.

In a study on social representations of pregnancy in primiparous women, authors state that the social representation of pregnancy can also be anchored in positive and negative reactions and that it highlights an ambivalence of feelings inherent to pregnancy, in which anxiety about the new is present, supporting the reports presented in this study by trans men^(20)^.

### C2) Family relationships, loneliness and lack of support

The meanings about family relationships established by trans men during pregnancy are represented by “harmonious relationships”, “lack of knowledge” and moments of “happiness” between subjects who are considered, by them, family members. These perceptions are crossed by expectations regarding transmasculine pregnancy in accordance with an idea of gender “detransition” or “retransition”. The reproduction of an idea of “detransition” by family members stands out, in which these men would once again recognize themselves as the gender designated at the time of their birth and play corresponding social roles by experiencing pregnancy, which is understood as an exclusively cisgender and heterosexual event^(3)^.

It appears that the social representations of these trans men regarding family relationships during pregnancy are constituted by a social affirmation of this “pregnant” and “masculine body”, explaining pregnancy as a possible event and breaking with stereotypes constructed about this phenomenon, such as the elaborate idea that transgender people cannot have mutual emotional and sexual relationships as well as exercise the right to parenthood through pregnancy.

This moment was also represented as a “lonely experience”, since, as they believed in the risks of transphobic violence in public spaces due to their condition as a pregnant man, “social isolation” becomes a reality throughout the gestational process, and can extend until the postpartum period, generating significant trauma and repercussions on mental health. Another important aspect is supportive relationships with their partner during pregnancy. Among the three reports that established same-sex relationships between gay men (one trans man and the other cisgender), two of these are anchored by a “toxic masculinity” reproduced by cisgender partners. These relationships were signaled by a lack of support, and, when pregnant men felt supported, this event was related to the “financial” issue, understood as an “obligation” and being insufficient for pregnant trans men^(3)^.

It is clear that same-sex and supportive relationships during pregnancy were represented by a “negative and conflict-ridden” relationship that permeates historical, social and cultural constructions about gender roles attributed to pregnant people. In this case, trans men occupy the role of “caregivers”, and their cisgender partners reproduce behaviors under the lack of commitment to the effective exercise of parenthood or even lack of knowledge and insecurity to recognize and take over their role in raising children in the face of clashes with socially established family standards.

Three reports stand out in which relationships occurred with transcentric couples, in the case of a configuration in which two transsexuals and transvestites have an affective-sexual relationship. They were marked by “mutual respect and support” and permeated by respect for their gender identity and pregnancy as an event that constituted their masculinity, including the dynamics among family members. It is also observed that trans men represent pregnancy as a “lonely” and “oppressive” moment. Loneliness was the overarching theme that permeated the experiences, causing them to construct coping strategies to manage their feelings. Some speeches reinforce that pregnancy was marked by a deep feeling of “not recognizing oneself” and “lack of connection with a developing fetus”, which is in line with a study in which one of the participants stated that he “hated being pregnant” and described profound loneliness during pregnancy^(3,21)^.

### C3) Senses and experiences during healthcare

This category relates these subjects’ social representations about pregnancy to fear of the parturition process, which is the moment when healthcare is provided during labor and birth, being anchored by the socially widespread perception that they are not biologically capable of performing a vaginal delivery. This fact may be related to the occurrence of obstetric and transphobic violence in institutions marked by gender specificities, such as “maternity wards”, in addition to exposure of these “abject” bodies that are not recognized as possible to “gestate” among healthcare professionals at the time of childbirth^(8)^.

It was observed that the content of social representations of healthcare during pregnancy was also linked to the preference for prenatal care in a private healthcare service with an obstetrician doctor and, consequently, through cesarean childbirth instead of vaginal delivery and obstetric care offered by the Brazilian Health System (SUS - *Sistema Único de Saúde*) services, in this case, seen as a place with a greater possibility of violence occurring during a “magical and challenging” moment in their life. Studies have shown that there are factors that influence the choice of childbirth route, such as psychocultural, socioeconomic, demographic aspects and, especially, medical interference, which sometimes contribute to a targeted decision. Sometimes, these factors converge to denaturalize vaginal delivery and praise the choice for cesarean birth; therefore, the decision to opt for childbirth does not always follow the clinical eligibility criteria and pregnant people’s initial wishes, compromising their autonomy in this process^(22,23)^.

In the context of obstetric care, the persistence of the technocratic care model in healthcare services attributes responsibility to the institution and authority to medical professionals over pregnant people through the “control” of their bodies, evidenced by the passivity of users’ choice in the face of the doctor, seen as a “figure of knowledge”, in which they place their trust^(24)^. There is a lack of preparation of pregnant men for labor and childbirth, with insecurity, anguish and fear present at this time. It is important that the health team accepts their demands, seeks to understand how care was provided during prenatal care and outlines the strategies that best suit these individuals, considering their specificities. It is noteworthy, therefore, that nurses play a fundamental role during this process, which ranges from prenatal care and continues through to the postpartum period^(25)^.

In our study, among the seven trans men who underwent prenatal appointment, only one was followed up by a nurse who worked at a Family Health Unit (FHU), including situations of transphobic violence in prenatal care, such as the insistence on requesting the civil registry name to fill out forms during care, not guaranteeing respect for the use of social names, when not rectified, and compromising pregnant men’s adherence to the service. The condition of socioeconomic vulnerability that led this participant to seek care in the SUS stands out.

In this context, the social representations constructed about obstetric violence that emerge throughout reports of trans men in this study are assimilated with lack of respect for their social name during prenatal care appointments, disrespect for male gender identity during the pregnancy-puerperal cycle, delegitimization of pregnancy based on cisgender stereotypes by healthcare professionals, denial of access to public or private healthcare services intended only for cisgender pregnant women, denial of pregnant men’s right to sign the documents made available by health facilities at the time of childbirth as a “father” and difficulty in sitting in preferential seats for pregnant people identified in the country’s various public transports.

Despite this, positive experiences were reported during healthcare, characterized by clinical meetings that provided privacy, naturalization of trans pregnancy, recognition of their parenthood and absence of humiliating attitudes^(26)^. Furthermore, pregnancy was assimilated as an event that enabled “self-knowledge”, “positive and negative changes” and “pedagogical” together with the recognition of the power of the transmasculine body to gestate and make this phenomenon a constituent of its masculinity. These aspects are directly related to the ability that pregnancy provided to bring important reflections to everyone who had the opportunity to share experiences with this group during pregnancy, whether friends, family or healthcare professionals, and who previously did not have access to these realities, acting as a mechanism for changing perspectives and social transformation.

### C4) Body and emotional changes and strategies for hiding pregnancy

This category shows that the social representations of pregnancy among participants in this study are associated with the physical and emotional changes brought about by the gestational process, which sometimes appear simultaneously, having an influence on the experience of these subjects. Such changes are related to how these people perceive their body during pregnancy, through a process of non-recognition of their image as a man and reinforcing gender standards and roles attributed to pregnancy as “*a more feminine thing a trans guy can do*” (I1).

In this subjective conflict, the use of gender affirmation technologies, such as “binder”, “micropore tape”, coats, warm blouses and loose clothing, is important as a strategy for maintaining stereotypes constructed under the logic of hegemonic masculinity during pregnancy, hiding the physical characteristics provided by pregnancy and socially considered “feminine”, contributing to promoting a “passability” of this body in public spaces, generating safety and impacting their quality of life.

It is clear how difficult it is for trans men to deal with bodily changes, especially those that are close to the symbol of femininity, such as breast enlargement, increased “pregnant” belly and weight gain, impacted by the recommendation to interrupt masculinizing hormones during this period of their lives and interfering with the process of gender affirmation, through the reduction of characteristics socially seen as masculine in the materiality of their bodies.

Only one report in this study says they used hormones during pregnancy, generating “increased hair” in children after birth. There is a lack of literature on the effect of gender affirmation through the use of testosterone on the reproductive function of trans men. Sometimes, healthcare professionals use data resulting from research carried out with cisgender women, inferring recommendations for healthcare for this group^(27)^. These data support one of the reports presented in this research, in which the idea of using testosterone and not menstruating, due to the effects of the medication, would have no risk of becoming pregnant; however, testosterone is not a contraceptive and, despite being used to promote “masculine” characteristics, some of these men maintain their reproductive functions adequately, and the use of internal or external condoms or other contraceptive methods is recommended^(28)^.

With regard to breastfeeding, whether or not to undergo masculinizing mastectomy surgery before becoming pregnant was the main factor that affected the decision surrounding this experience. Furthermore, the fact of recognizing breastfeeding as a strategy to provide nutrition had significant influences that permeate feelings of “leaving oneself aside” to fulfill this role, which would be “a greater good” for children, and this practice can be given new meaning throughout the gestational process. In this study, of the seven trans men participating, five of them chose to breastfeed. Some reports available in the literature mention that many feel comfortable breastfeeding in public spaces, this being a political act^(29)^.

It is necessary to use other infant feeding options for children of trans men who do not choose to breastfeed, such as the use of milk banks or milk formulas, and it is the responsibility of professionals to communicate during prenatal care about the different options, not putting pressure on users and respecting their autonomy^(29)^. In this context, it is essential for professional nurses, in the exercise of their role as caregiver and educator, to use health education as a strategy to discuss with users the importance and different possibilities of infant feeding, considering their concrete reality.

With the increase in breasts, it is clear that these men often use strategies to hide this part of the body, through the use of “binder” or “micropore tape”; sometimes, the use becomes so intense that it ends up being compared to “torture”, due to the pain and discomfort caused. The use of these technologies results in frequent isolation, due to a context of transphobic violence that can last until the postpartum period^(30-33)^. For some trans men, using these strategies to hide characteristics typical of pregnancy were part of their experience, in order to increase feelings of security when going out in public, with use of coats, warm sweaters and loose clothing being the main ones, in an attempt to not promote the visualization of the changes brought about by pregnancy. Studies show that trans men who went through this experience noticed that their pregnancies were often identified as obesity, and were generally easier to disguise with clothing than the breast itself^(18,29)^.

From that same study, those who pass as a cisgender man reported being perceived as an obese man and never as a pregnant woman. In contrast, other trans men became publicly visible while pregnant, however some participants feared a greater likelihood of transphobic violence^(26)^. This data supports one of the reports mentioned here, in which the fact of being pregnant has been something positive, generating feelings of “happiness when seeing the belly grow”, without affecting their masculinity.

### Study limitations

This study faced challenges due to limitations associated with the restricted number of participants, as it focused on a specific and difficult-to-reach population. These difficulties were exacerbated during the COVID-19 pandemic period, especially due to the socioeconomic vulnerability of the context. Furthermore, there were obstacles in the inclusion of transgender men in the study, as some of them did not respond to the main researcher’s contacts or did not express interest in participating in the research conducted by a cisgender person.

### Contributions to health, nursing or public policy

This study reinforces the importance of advanced nursing practice in the context of prenatal, obstetric and postpartum care, starting from the perspective of comprehensive care, equity in access to services and respect for differences, highlighting sexual orientation and gender identity as Social Determinants of Health. Therefore, they can support future reflections in the field of study on transmasculinities, sexual and reproductive rights with repercussions on the formation of public health policies for an equitable, comprehensive, welcoming and resolute practice for the trans population, breaking stigmas and transphobic processes.

## FINAL CONSIDERATIONS

Social representations about pregnancy among trans men involved a wide field of meanings, in which their efforts were articulated in “accepting” pregnancy by “leaving oneself aside” and choosing to continue the gestational process, being represented as a “lonely experience”. Healthcare was linked to the preference for prenatal care in a private service and, consequently, through cesarean childbirth. Physical and emotional changes brought about by the gestational process were related to how these people perceive their bodies during pregnancy, through a process of non-recognition of his image as a man and reinforcing gender standards and roles attributed to pregnancy as something feminine.
