# Supplementary material for: Social Representations of pregnancy among trans men
Source: Rev Bras Enferm. 2025 Jan 10;77(Suppl 2):e20240094. doi: 10.1590/0034-7167-2024-0094 (PMC11726815; doi:10.1590/0034-7167-2024-0094)
Supplement: Translation

# Translation: Representações Sociais da gestação entre homens trans

**DOI:** 10.1590/0034-7167-2024-0094pt

**Authors:** Danilo Martins Roque Pereira, Ednaldo Cavalcante de Araújo, Sheyla Costa de Oliveira, Anderson Reis de Sousa, Mariana Mercês Mesquita Espíndola, Milka Gabrielle de Lira Nóbrega West, Marclineide Nóbrega de Andrade Ramalho, Joecio Cordeiro Cardoso

**Affiliations:** I Universidade Federal de Pernambuco. Recife, Pernambuco, Brasil; II Universidade Federal da Bahia. Salvador, Bahia, Brasil; III Centro Universitário FMABC. Santo André, São Paulo, Brasil

**Keywords:** Teoria Social, Minorias Sexuais e de Gênero, Gravidez, Cuidado de Enfermagem, Educação em Saúde

## Abstract

**Objetivo:**

conhecer os significados e as experiências da gestação entre homens trans à luz da Teoria das Representações Sociais.

**Métodos:**

trata-se de estudo qualitativo, descritivo e exploratório, realizado com homens trans selecionados por conveniência e disponibilidade. A produção de dados ocorreu de setembro a outubro de 2021, pela plataforma *Google Meet^®^*, a partir de entrevistas com roteiro semiestruturado. A análise textual lexicográfica das entrevistas foi pelo método Reinert e instrumentalizada pelo IRAMUTEQ, versão 7.0.

**Resultados:**

as representações sociais da gestação envolveram um amplo campo de significados, em que foram articulados esforços em aceitar a gravidez oportuna e solitária, medo do processo de parturição e influência das mudanças físicas e emocionais.

**Considerações finais:**

o estudo reforça a importância de uma prática avançada de enfermagem na assistência ao ciclo gravídico-puerperal, partindo da perspectiva de uma integralidade no cuidado, da equidade no acesso à serviços e do respeito às diferenças.

## INTRODUÇÃO

Na sociedade ocidental, a gestação paterna é geralmente vista como uma possibilidade “improvável” para os “homens trans”, pois a reprodução é considerada impossível para indivíduos concebidos através da ideia de abjeção. Isso resulta em uma condição denominada “esterilidade simbólica”, caracterizada pela negação do direito de escolha para a reprodução e para exercer a parentalidade, seja ela biológica ou não^(1)^.

Nesse cenário, homens trans que passaram pela gestação ou estão em processo gestacional conferem significados aos eventos da gestação, parto e amamentação como elementos intrínsecos à construção de sua masculinidade. Isso ocorre mesmo sendo indivíduos que possuem vagina, útero e ovários, desafiando, assim, as normas reprodutivas cisheteronormativas^(2,3)^.

Pesquisas evidenciaram que homens trans que vivenciaram a gestação apresentam demandas específicas relacionadas aos efeitos do uso de hormônios, às intervenções cirúrgicas e às mudanças nas percepções corporais ocorridas durante esse período^(4)^. Aliado a isso, a transfobia tem promovido uma “expulsão” desse público dos serviços da saúde, a partir do não respeito ao nome social, uso inadequado dos pronomes de tratamento, questionamentos inapropriados, déficit de informações que contemplem suas especificidades de saúde, entre outros, configurando-se, também, barreiras de acesso à saúde integral^(5,6)^.

No contexto do cuidado, na perspectiva da promoção à saúde, a educação em saúde emerge como um recurso a ser utilizado pelo enfermeiro no desempenho do papel de educador em saúde, com a capacidade de potencializar a reflexão dos homens trans em processo de gravidez acerca de suas representações oriundas de suas vivências, propiciando a criticidade e a capacidade de compreensão de fragilidades e habilidades de superar as dificuldades que ora lhes possam apresentar nessa fase de vida^(7,8)^.

Nesse sentido, investigar as representações sociais de homens trans que engravidaram ou estão passando pela gestação possibilita uma compreensão de suas interpretações e informações sobre esse fenômeno sob a perspectiva do senso comum. Isso, por sua vez, permite ao enfermeiro desenvolver práticas de saúde e se engajar na luta contra a transfobia institucional^(8)^. Entende-se que a Teoria das Representações Sociais (TRS) oferece um arcabouço teórico para compreender a vida cotidiana de grupos específicos, possibilitando a criação de um conhecimento prático ao ser integrado às experiências, que incluem contexto histórico, cultural e espacial, e ao orientar as comunicações e comportamentos dos indivíduos^(9)^.

Assim, diante do exposto, este estudo teve como pergunta condutora: quais os significados e as experiências da gestação entre homens trans?

## OBJETIVO

Conhecer os significados e as experiências da gestação entre homens trans à luz da TRS.

## MÉTODOS

### Aspectos éticos

O projeto de pesquisa foi submetido ao Comitê de Ética em Pesquisas (CEP) do Centro de Ciências da Saúde (CCS) da Universidade Federal de Pernambuco (UFPE), sob Parecer n^o^. 4.862.503, aprovado no ano de 2021, em atendimento à Resolução n^o^. 466/12 do Conselho Nacional de Saúde (CNS), que regula as diretrizes e normas que tratam de pesquisa com seres humanos. Após a aprovação do projeto de pesquisa no CEP/UFPE, a produção de dados empíricos foi iniciada. A identificação dos participantes foi anonimizada, utilizando pseudônimos autodeclarados no momento da coleta de informações.

### Tipo de estudo

Estudo qualitativo, descritivo e exploratório, estruturado nas diretrizes do *Consolidated criteria for Reporting Qualitative research* (COREQ) e ancorado pela TRS de Serge Moscovici e seguidores. Com vistas a acessar o conhecimento sobre a realidade de um determinado grupo social, a TRS permite à enfermagem guiar o cuidado e conduzir a assistência em saúde a partir do reconhecimento de suas características e especificidades^(10)^.

### Cenário do estudo

A pesquisa foi conduzida em nível nacional, envolvendo indivíduos vinculados a instituições de destaque para a população de Lésbicas, Gays, Bissexuais, Travestis e Transexuais (LGBT) no Brasil. Essas instituições incluem a Aliança Nacional LGBTI+, o Ambulatório LGBT Patrícia Gomes, em Recife, Pernambuco, e a Coordenação de Saúde LGBT do Estado de Pernambuco. A seleção desses locais de pesquisa seguiu critérios intencionais de amostragem, visando incorporar características essenciais para desenvolver a pesquisa e abranger a população de interesse.

### Fonte de dados

Os participantes do estudo foram homens trans que atenderam aos seguintes critérios: 1) de inclusão: homens trans gestantes ou que foram gestantes e com idade igual ou superior aos 18 anos de idade; 2) de exclusão: homens trans gestantes ou que passaram por uma gestação, que tinham deficiência visual total e/ou auditiva, visto a inabilidade do pesquisador da compreensão em Libras e Braile, e com problemas de saúde que prejudiquem a compreensão e participação do estudo ou que não possuíam recursos tecnológicos para a realização da pesquisa (computador, celular, *tablet, internet*, entre outros), considerando o contexto de pandemia. A seleção dos participantes se deu por conveniência e disponibilidade para serem entrevistados. A escolha dessa técnica decorreu da necessidade em reunir a população de interesse, por possuírem características peculiares ou de difícil identificação^(11)^. Foram incluídos sete participantes por inserção progressiva, a partir da saturação teórica das respostas às entrevistas semiestruturadas^(12)^.

### Coleta e organização dos dados

Para a coleta das informações, que ocorreu de setembro a outubro de 2021, foi utilizada a técnica de entrevistas individuais semiestruturadas, por meio de chamada telefônica ou pela plataforma *Google Meet^®^*. Um roteiro auxiliou a condução da entrevista e reuniu questionamentos dos aspectos relevantes ao objetivo do estudo. Foi composto pelas questões norteadoras: 1. Fale-me sobre como foi a sua gestação. 2. Fale-me sobre as modificações (corporais, sociais, emocionais, psicológicas, entre outras) do início até o fim da gravidez. 3. Fale-me como foi sua assistência à saúde prestada pela equipe de saúde para o acolhimento e cuidado durante a gestação. 4. Você conhece outros homens trans que engravidaram ou que estejam grávidos?

Posteriormente, foram agendados os encontros com o público-alvo por meio de chamada telefônica ou aplicativos de troca de mensagens, como *WhatsApp^®^, Instagram^®^, Facebook^®^* ou e-mail, a partir da disponibilidade do participante de pesquisa. Foi recomendado ao participante, no momento da entrevista, permanecer em ambiente privado e silencioso, critérios necessários para que houvesse o mínimo de interferência externa. Os entrevistados foram informados sobre o objetivo da pesquisa pelo Termo de Consentimento Livre e Esclarecido (TCLE) e sobre a necessidade de assinatura do Termo de Autorização de Uso de Imagem e Depoimento, disponibilizado pela plataforma *Google Forms^®^*. Além disso, foi aplicado um formulário para preenchimento de dados de caracterização dos participantes, disponibilizado antes das entrevistas e pelas principais redes sociais (*Facebook^®^, Instagram^®^* e *WhatsApp^®^*). As entrevistas individuais *online* foram consentidas e gravadas em vídeo por, no máximo, duas horas, pelo recurso disponibilizado pela plataforma *Google Meet^®^.* Após cada transcrição, encaminhou-se aos entrevistados para validação de conteúdo, dando-lhes a oportunidade de acréscimo ou retirada de informações, caso necessário. Foi solicitado que as câmeras, no momento das entrevistas *online*, estivessem ligadas, de modo que as pausas discursivas e entonações fossem captadas pelo pesquisador, assim como as expressões faciais, gestos e outras linguagens corporais.

### Análise dos dados

As informações empíricas produzidas pelas entrevistas individuais foram submetidas à análise textual por meio do método Reinert de classificação de segmentos de texto (ST), instrumentalizada pelo *software* gratuito *Interface de R pour les Analyses Multidimensionnelles de Textes et de Questionnaires* (IRAMUTEQ), versão 7.0. Foi utilizada a Classificação Hierárquica Descendente (CHD) como técnica de tratamento das informações^(13)^.

## RESULTADOS

O universo de significados das representações sociais de sete homens trans sobre a gestação está descrito abaixo, fundamentando os questionamentos propostos nesta pesquisa, traduzindo o “senso comum” deste grupo. Para uma maior riqueza dos dados, o Quadro 1 apresenta a caracterização de acordo com o perfil do ciclo gravidez-parto dos homens trans, de modo a visualizar o perfil desses e melhor situar a amostra, uma vez que o objeto de qualquer representação social se define a partir do fenômeno e do grupo escolhido^(14)^. Esses foram identificados por pseudônimos autodeclarados no momento das entrevistas.

**Quadro 1:** Caracterização do perfil do ciclo gravidez-parto dos homens trans participantes deste estudo. Recife, PE, Brasil, 2022

	Como engravidou	Fez uso de hormônio masculinizante durante a gestação	A gravidez foi planejada	Tentativas de interrupção da gestação	Tipo de parto	Realiza/realizou consultas pré-natal	Em caso de “sim” na pergunta anterior, qual profissional realizou seu pré-natal	Número de filhos(as)
**E1**	Relação sexual pênis-vagina	Não	Não	Não	Normal	Sim	Médico	1
**E2**	Relação sexual pênis-vagina	Sim	Não	Não	Cesário	Sim	Médico	1
**E3**	Relação sexual pênis-vagina	Não	Não	Não	Cesário	Sim	Médico	1
**E4**	Relação sexual pênis-vagina	Não	Não	Não	Cesário	Sim	Médico	1 biológico e 2 não biológicos
**E5**	Relação sexual pênis-vagina	Não	Sim	Não. Relata aborto retido anterior.	Cesário	Sim	Médico	1
**E6**	Relação sexual pênis-vagina	Não	Não	Não. Relata aborto espontâneo anterior.	Pretende-se parto normal.	Sim	Enfermeiro	0
**E7**	Relação sexual pênis-vagina	Não	Não	Não	Normal	Sim	Médico	1

O *corpus* de análise deste estudo foi composto por sete textos, submetidos à análise por obtenção da CHD, dividido em 1.172 ST, relacionando-se 3.883 palavras, com 41.094 ocorrências. A CHD obteve 92,75% do total de ST, correspondente a 1.087 ST, gerando quatro classes. A seguir, as classes estão descritas seguindo-se a ordem de partição e de proporção que representam em relação ao *corpus* total.

A classe 1 (Quadro 2), “Descoberta da gestação e suas implicações”, corresponde a 41,12% dos ST, relacionando-se ao momento em que homens trans descobrem a gestação e os desdobramentos desse acontecimento em suas vidas. O significado dessa classe diz respeito a um momento em que os sinais da gestação são confundidos com comorbidades diversas, atribuindo a confirmação da gravidez não esperada à sensação de “desmoronamento”, “insegurança” e “sem saber o que fazer”, além do abortamento como alternativa de interrupção. Destaca-se o relato do E4, que discorre sobre a ocorrência de um “estrupo corretivo” e que teve como consequência uma gestação, sendo esse o único relato que evidencia este fato, portanto, imprescindível nas reflexões sobre as implicações para o cuidado.

**Quadro 2:** Classe 1: Descoberta da gestação e suas implicações. Recife, PE, Brasil, 2022

Identificação	Depoimento
**E1 - Bernardo**	[...] *quando veio a notícia, foi tipo a sensação, sei lá, como se tivesse dentro de uma casa e ela tivesse terminado de desmoronar* [...] *porque a primeira coisa que eu pensei é: “O que é que eu vou fazer?”. E a primeira reação do outro pai foi: “Ah, vamos tirar!”. Ele não chegou pra dizer assim: “E aí? Tu vais querer?”* [...] *e eu me culpava por não ter sido mais responsável, e aí vem o fato de que eu tinha acabado de sair de uma depressão, o relacionamento ruim, e estava com medo de perder o emprego, porque eu tinha acabado de entrar na empresa* [...].
**E2 - Iago**	[...] *a médica olhou para minha cara e falou: “Posso te fazer uma pergunta? Tem a possibilidade de você estar gestante?”. Eu disse: “Olhe, eu transo sem camisinha, mas eu tomo hormônio e faz anos que falam pra mim que não posso ter filho porque sou estéril”. Eu disse: “Olha, eu fui ao hospital lá da minha cidade; ele disse pra mim que eu estava com uma bactéria muito forte no estômago e passou até o medicamento pra mim dizendo que isso é gases”. Ela disse: “Isso não é gases, não, se isso não for uma criança, eu deixo de ser médica!”. Quando ela disse isso, minha cara caiu no chão* [...].
**E3 - Coutinho**	[...] *antes da gravidez, já estava a dois meses sem hormônios, usava há dois anos Deposteron. Não foi planejada, mas era desejada. Minha noiva não estava fazendo o uso de hormônios e nem bloqueadores também.* [...] *eu descobri uma semana antes da minha mastectomia ... e não foi um momento feliz* [...] *foi um momento que eu senti que eu não era mais dono do meu corpo, sabe? ... eu não estava preparado para a gestação. Naquele momento, eu estava me preparando pra realizar outro sonho que era o da mastectomia ... a minha saúde mental foi para zero* [...].
**E4 - Lessa**	[...] *sobre a gestação, eu nunca imaginei ser pai ou ser mãe, né? Na época, ainda era lido como mãe, mas eu nunca tinha imaginado essa possibilidade, e as coisas aconteceram de uma maneira que eu não esperava. Sofri o que hoje a gente conhece como estupro corretivo... e, durante a violência, o agressor dizia que ele ia me mostrar o que era ser um homem de verdade e em alguns momentos ele dizia que ia me fazer ser mulher de verdade* [...].
**E5 - Brandon**	[...] *a gestação do nosso filho foi extremamente planejada* [...] *o planejamento, ele ocorreu de uma maneira muito autônoma, está bem?* [...] *eu e minha companheira decidimos em algum momento que iria passar por esse processo, e aí a gente começou a pesquisar* [...] *e vimos que nós precisaríamos parar de tomar hormônio no processo e paramos* [...].
**E6 - Matheus**	[...] *o único ponto que ficava na minha cabeça, se conseguiria gestar ou não, não tinha a ver comigo mesmo, eu e meu corpo. Era mais uma relação eu com a sociedade, como que eu ia lidar com a resposta social* [...] *se eu ia conseguir lidar com isso* [...].
**E7 - Azevedo**	[...] *a gestação não foi uma coisa planejada, foi bem no susto mesmo. No começo, foi até um baque pra mim. Teve pensamentos de início até de tirar* [...] *foi em uma temporada em que eu tive que dar uma pausa nos hormônios e acabou acontecendo* [...] *quando eu fui descobrir que eu estava grávido* [...] *já estava esperando meu filho já tinha três meses já e eu não tinha nem noção* [...] *no começo, eu fiquei meio assustado, principalmente por conta do medo das coisas que eu ia passar, preconceito* [...].

A classe 2 (Quadro 3), “Relações familiares, solidão e falta de apoio”, corresponde a 27,97% dos ST, relacionando-se como os homens trans estabeleceram seus vínculos familiares durante a gestação, reflexões sobre o apoio por parte do companheiro (ou da companheira), questionamentos sobre a gestação sobre “de quem seria o filho”, atribuição à vivência da gestação enquanto um momento de “privação” e “solidário”.

**Quadro 3:** Classe 2: Relações familiares, solidão e falta de apoio. Recife, PE, Brasil, 2022

Identificação	Depoimento
**E1 - Bernardo**	[...] *ficaram extremamente felizes* (com a gestação), *e, na época, muitos ainda não me não me viam como o Bernardo, e me chamava com o nome antigo, então ficaram felizes porque era mais uma mulher da família tendo um filho* [...] *mas sempre nessa visão de que agora que vai ter um filho talvez volte atrás na transição, que era uma esperança que o pessoal tinha, entendeu? Até hoje tem gente que* [...].
**E2 - Iago**	[...] *tive uma raiva muito grande do pai da minha filha e acabei voltando para o hospital* [...] *eu disse: “Quer fazer o exame de DNA, a gente faz quando ela nascer* [...] s*e quiser fazer agora, pode fazer. A partir do quarto mês de gestação, pode fazer exame de DNA!* [...] *eu quero porque você está duvidando da minha palavra. Eu não tenho porque dizer que ela era a tua filha, não”* [...].
**E3 - Coutinho**	[...] *na minha cabeça, eu não tinha condições de ser a casa de um bebê, porque nem eu mesmo estava me entendendo como ser humano, entende?* [...] *eu não conseguia olhar pra mim e pensar que aquilo estava sendo bonito, entende?* [...] *eu só pensava que eu queria que passasse logo, sabe?* [...] *é tudo solitário e a gente passa muito tempo com a gente mesmo* [...].
**E5 - Brandon**	[...] *quando souberam* (a família), *ficaram muito felizes. Eu cheguei a contar pra minha mãe que eu estava passando pela gestação* [...] *só que aí vieram cobranças do tipo “você vai tirar a barba, né?”, tipo, já que você está passando pela gestação, então você vai destransicionar* [...] *eu expliquei que não e que, na verdade, é transicionar que me mostrou a gestação enquanto o processo possível. Rolou muita falta de conhecimento nesse processo* [...] *de não compreender a gestação como a possibilidade da minha masculinidade* [...].
**E6 - Matheus**	[...] *e então eu sou um pouco distante da minha família por parte de mãe, mas, quando eu contei pra eles, não teve nenhum questionamento. Ninguém perguntou absolutamente nada, estou feliz. O meu pai* [...] *eu fui falar pra ele: “Ó, talvez esteja chegando mais um novo membro da família e o senhor vai ser vovô de novo”. Aí ele falou assim: “Como assim? Quem é, quem é?”, aí eu falei: “Como assim de quem é, mano? Está doido? É meu!”* (risos). *E aí ele falou: “Mas sério? Eu não achava que vocês transavam desse jeito”* [...] *deu uma bugada na cabeça dele* [...].
**E7 - Azevedo**	[...] *as violências que vivenciei durante a gestação me afetaram bastante, porque eu saía na rua mesmo só porque precisava trabalhar. Fora isso, por tanto medo dessas coisas, de apanhar de novo ou dos preconceitos que eu já estava sofrendo, das pessoas ver a barriga e não entender* [...] *eu me privei bastante, eu fiquei trancado dentro de casa a gestação inteira até depois que eu ganhei meu filho* [...].

A classe 3 (Quadro 4), “Sentidos e experiências durante a assistência à saúde”, corresponde a 19,96% dos ST e diz respeito aos sentidos construídos sobre as experiências durante a assistência à saúde, dificuldades durante o planejamento reprodutivo em uma relação transcentrada, incômodo no compartilhamento de espaços “femininos” durante a assistência pré-natal, negação de direitos ao gestante, reprodução de discursos transfóbicos por profissionais de saúde e acompanhamento pré-natal entre o público e privado. Evidencia-se, também, a gestação enquanto uma experiência de “autoconhecimento”, “de mudanças positivas e negativas” e “pedagógica”, a partir do reconhecimento das potencialidades de seu corpo e da compreensão da gestação enquanto um fenômeno que constitui sua masculinidade. O medo do momento do parto torna-se presente e relaciona-se à capacidade do corpo em ter um parto normal, mas, principalmente, à ocorrência da violência obstétrica e transfóbica em instituições de saúde, visto que a assistência ao ciclo gravídico-puerperal se materializa em locais pensados para o público feminino e cisgênero, e, no caso de gestações transmasculinas, esse aspecto pode se agravar.

**Quadro 4:** Classe 3: Sentidos e experiências durante a assistência à saúde. Recife, PE, Brasil, 2022

Identificação	Depoimento
**E1 - Bernardo**	[...] *como eu estava trabalhando, e no trabalho eu tinha plano de saúde, eu falei: “Vou pelo caminho mais fácil teoricamente”* [...] *botam muito terror na questão do parto pelo SUS, sabe? Do tratamento, e como é feito e tudo mais, e isso com mulheres cis, imagina um homem trans* [...] *e aí eu ficava com muito medo de como seria, sabe?* [...] *pra mim, um dos maiores defeitos do pessoal da saúde é claramente que eles não sabem como cuidar de um corpo trans, e aí meio que a gente tem que ensinar, sabe?* [...].
**E2 - Iago**	[...] *era algo que me incomodava muito estar num ambiente muito feminino* [...] *eu fiquei com muito medo, muito medo mesmo, eu chega me tremia* [...] *teve uma técnica de enfermagem que ... acordou de manhã e disse: “Eu não vou te chamar de “ele” não, você é “ela”! Que homem não engravida, você tem uma boceta”. Aí as meninas do quarto disseram “Nem liga!”. Aí ela disse: “Você vai ter uma criança, vai deixar a criança perturbada* [...] *você quer ser o que você não pode! Se enxerga. Você é uma mulher* [...] *tem uma buceta. Você não tem rola, não!”* [...] *aí a minha pressão já subiu. Eu já comecei a me sentir mal* [...].
**E5 - Brandon**	[...] *sobre a assistência por minha obstetra* [...] *o que eu vou ter são algumas barreiras institucionais ... por exemplo, ela só tinha um banheiro feminino no consultório dela. Só tinha um banheiro com a placa feminina. Então, assim, são coisas que muitas vezes a gente chama atenção pra pessoa que são microviolências simbólicas que vão me dizendo que aquele lugar ali não me pertence,* [...] *ela solicitou que eu tomasse uma determinada vacina durante o período da gestação e eu fui me dirigir a uma Unidade de Saúde da Família e, quando chegou lá, eu tive que basicamente passar por um interrogatório com um Agente Comunitária de Saúde, e ele não entendia que eu era um homem trans* [...] *e ele dificultou meu acesso a tomar aquela vacina ...* [...] *ele entendeu, por mais que eu estivesse repetindo pra ele, que eu estava gestando; ele achou que eu estava sendo uma fraude ali, né?* [...].
**E6 - Matheus**	[...] *na clínica da família* [...] *lá foi maravilhoso, assim a menina em nenhum momento me destratou, ela só ficou na dúvida, não entendeu direito tudo, e eles tiveram que fazer uma mudança, porque, no SUS, se você é do sexo masculino, você não pode entrar no protocolo na linha de cuidado de gestante, né? Então, eles tiveram que fazer uma mudança* [...] *eles mudaram o meu sexo pro feminino, mas o meu nome continuou da mesma forma, e aí o rapaz me disse que ele iria encaminhar um e-mail pra o responsável criador do “coisa do SUS”. Eu duvido muito, mas, assim, achei muito fofo de qualquer forma, é um cuidado deles, né? ... agora, o enfermeiro, especificamente, foi péssimo, foi horrível* [...] *ele não sabia se era eu que estava grávido, se era minha companheira, ele ficou bugadaço. Ele queria saber meu nome de registro de todas as formas possíveis, e eu não disse meu nome de registro, ele me trata só no feminino* [...].
**E7 - Azevedo**	[...] *eu tinha o físico já totalmente masculino, barba* [...] *e quando começou a barriga crescer, aí que foi um baque bem maior mesmo. Tive dificuldades para pessoas cederem lugares dentro do ônibus e eu trabalhei durante toda minha gestação. Bem no finalzinho mesmo, perto de ganhar, que eu ganhei a licença. Todos os dias quando eu pegava ônibus, teve dois episódios só que eu tive pessoas que cederam lugar para mim me sentar. Eu ia sempre em pé e sempre correndo risco* [...].

A classe 4 (Quadro 5), “Modificações corporais, emocionais e estratégias de ocultação da gestação”, corresponde a 10,95% dos ST, relacionando-se às mudanças físicas e emocionais decorrentes do processo gestacional e da influência que essas desempenham na vivência desses homens. São considerados nessa classe aspectos como aumento do desejo em realizar a cirurgia de mastectomia após a gestação, aumento da mama como um dos maiores desconfortos, uso do “*binder*” ou “fita micropore” e sutiã durante a gravidez, incômodo com a voz, uso de casacos de frio como estratégia de ocultação da “barriga de grávido”, inchaço e dor na cicatriz cirúrgica da mastectomia masculinizadora, não se sentir desejado sexualmente pelo parceiro e não reconhecimento de si mesmo diante da imagem refletida no espelho.

**Quadro 5:** Classe 4: Modificações corporais, emocionais e estratégias de ocultação da gestação. Recife, PE, Brasil, 2022

Identificação	Depoimento
**E1 - Bernardo**	[...] *o fato de eu estar grávido, o corpo querendo ou não muda, e querendo ou não* [...] *vamos dizer, assim, é a coisa mais feminina que um cara trans pode fazer... acho que o que mais gritou foi o peito* [...] *foi pra mim o pior, porque todo o resto foi muito tranquilo, porque eu sempre fui gordo, então engordar não foi um problema e eu lido muito bem com o fato de ter vagina* [...] *agora o peito, é o peito pegou valendo* [...] *foi bem complicado lidar e usar o peito depois pra amamentar o meu filho* [...].
**E2 - Iago**	[...] *o peito inchou de leite, então ficava uma coisa muito feia. Eu optei a usar o sutiã. Me incomodava* [...] *mas, por questão da minha filha, eu usava sutiã e dava de mamar ela nos cantos, e os outros ficavam olhando porque eu tinha barba* [...] *uma coisa que me incomoda até hoje é a voz.* [...] *a voz entrega tudo* [...].
**E3 - Coutinho**	[...] *aceitar as modificações, as disforias, todo dia tenho que entender que é passageiro, que vou voltar a ser o que eu era e que tudo isso por um bem maior, que é a saúde da minha filha* [...] *desde a primeira consulta, eu fui instruído a não usar mais a fita, o binder… nada que comprima os seios. Então, isso aí já me machucou bastante. Então, eu usava casaco e não aparecia quase nada ainda. Eu conseguia usar um top justo e não apertado* [...] *pra disfarçar um pouco com o casaco. Essa foi uma mudança muito dolorosa* [...].
**E5 - Brandon**	[...] *quando eu pensei na gestação, eu falei assim: “Olha, eu vou passar por esse processo”* [...] *eu tinha algumas coisas que eu estava disposto e outras que eu me tornei disposto, por exemplo a amamentação... o binder, eu sempre usei e, durante a gestação, eu tentei diminuir um pouco no final, porque eu sentia muita dor nas costas* [...] *e aí a tecnologia que eu utilizava é o velho casaco, né? Eu colocava casacos mais volumosos* [...] *era a maneira que eu me sentia mais confortável pra sair na rua sem muitas vezes ser identificado* [...] *inclusive era uma forma de resguardar a minha segurança, porque era muito comum quando as pessoas percebiam que eu era um homem trans, sabe? Eu recebia olhares de repressão* [...].
**E6 - Matheus**	[...] *eu estou lidando muito bem com as mudanças, e eu estou gostando de ver minha barriga crescer, estou feliz com isso* [...] *e em nada está afetando a minha masculinidade. Eu estou bem como o homem que eu sou. Eu sei as possibilidades que o meu corpo é capaz de fazer e isso não me faz menos homem* [...].
**E7 - Azevedo**	[...] *eu sempre tive uma disforia em relação a isso, principalmente por conta dos seios, e foi uma coisa que antes já me incomodava bastante e quando na gestação ficou bem maior ainda* [...] *a fita micropore, isso me prejudicou bastante* [...] *eu usava o dia todo, sempre que tinha que sair para alguma coisa assim, eu só tirava quando chegava em casa, na hora de dormir e tomar banho, essas coisas* [...] *e eu usava e me sentia bem melhor para sair e me apresentar fiscalmente* [...] *eu meio que não aceitava meu corpo mudar com aquilo, não aceitava aquilo dentro de mim* [...] *aí comecei a ficar mais tempo ainda com blusa de frio, vestindo roupas largas, roupas grandes para esconder o máximo possível que eu pudesse* [...].

## DISCUSSÃO

### C1) Descoberta da gestação e suas implicações

Observa-se que as representações sociais do impacto da descoberta da gravidez entre homens trans deste estudo e que engravidaram de maneira não esperada se ancoraram a sentimentos de “insegurança”, “desmoronamento”, “falta de responsabilidade” e “sem saber o que fazer”, atravessados pelo “medo da resposta social” diante de expectativas e aceitabilidade sobre um corpo transmasculino grávido. É possível considerar que essa categoria se associa, em alguns contextos, à ideia construída e dissipada culturalmente pela sociedade ocidental sobre a gestação enquanto fenômeno biológico, feminino e cisgênero. Neste caso, a “gestação cisheteronormativa” aparece como objetivação desta realidade e representa um caráter universal e homogêneo perante homens e mulheres, tida como o modelo da aceitabilidade sobre conceber uma criança, sobretudo a capacidade de dar à luz enquanto característica fundante da feminilidade sustentada por construções sociais de gênero, evidenciando papéis pré-estabelecidos socialmente, a exemplo das noções sobre maternidade e paternidade.

Percebe-se que os conteúdos das representações sociais sobre o impacto da descoberta da gestação entre homens trans envolvem um amplo campo de significados, em que são articulados os esforços deles em “aceitar” a gravidez oportuna por meio de um processo individual e subjetivo de empoderamento ao “deixar-se de lado”, ao optar pela continuidade do processo gestacional a partir do reconhecimento desse fenômeno como uma potencialidade de seu corpo, assumindo os riscos no enfrentamento das situações de violências transfóbicas e os desafios intrínsecos ao ciclo gravídico.

O estupro corretivo pode aparecer como mecanismo pelo qual homens trans estão vulneráveis e, consequentemente, sujeitos a engravidarem, que, nesse caso, caracteriza-se como uma violência sexual na qual o abusador objetiva “corrigir” a orientação sexual ou identidade de gênero da vítima, como apresentou-se um dos casos deste estudo. Ressalta-se que o estupro é um crime, previsto no Código Penal Brasileiro, em seu Artigo 213, na redação dada pela Lei nº. 12.015 de 2009^(15)^.

Questionamentos em favor ou contra as práticas abortivas se fazem presentes entre profissionais de saúde, mesmo quando se trata do aborto legal^(16)^. Na perspectiva das representações sociais, a condenação das práticas abortivas se relaciona com padrões morais, sociais e culturais, atravessados pela religião e pela legislação^(17)^. No entanto, neste estudo o abortamento aparece como uma alternativa viável para a manutenção do *status quo* promovido pela família tradicional e dos pilares reprodutivos que têm como fundamento a cisheteronormatividade.

Estudos já demonstram relatos de aborto espontâneo e aborto provocado entre homens trans e que se relaciona a diversos fatores; portanto, é preciso reconhecer a necessidade que os serviços de saúde lhes garantam o manejo adequado e humanizado às situações de risco e à assistência integral. A violência e o preconceito sofridos nos serviços de saúde por pessoas que optam pelo abortamento não são direcionados de forma restrita aos homens trans, mas são uma realidade que violenta o direito de escolha de homens e mulheres que engravidam^(18,19)^.

Em estudo sobre representações sociais da gravidez em mulheres primigestas, autores afirmam que a representação social da gravidez pode estar ancorada, também, em reações positivas e negativas e que evidencia uma ambivalência de sentimentos próprios da gestação, em que a ansiedade perante o novo está presente, corroborando os relatos apresentados neste estudo por homens trans^(20)^.

### C2) Relações familiares, solidão e falta de apoio

Os significados sobre as relações familiares estabelecidas por homens trans durante a gestação são representados por “relações harmônicas”, “falta de conhecimento” e momentos de “felicidade” entre sujeitos que são considerados, por eles, integrantes da família. Essas percepções são atravessadas por expectativas sob a gestação transmasculina em concordância a uma ideia de “destransição” ou “retransição” de gênero. Destaca-se a reprodução de uma ideia de “destransição” por familiares, em que esses homens voltariam a se reconhecer com o gênero designado no momento de seu nascimento e desempenhar papeis sociais correspondentes por vivenciar a gestação, que é entendida como um acontecimento, exclusivamente, cisgênero e heterossexual^(3)^.

Verifica-se que as representações sociais desses homens trans sobre as relações familiares durante a gestação são constituídas por uma afirmação social deste “corpo grávido” e “masculino”, explicitando a gestação enquanto um evento possível e rompendo com estereótipos construídos sobre esse fenômeno, a exemplo da ideia elaborada de que pessoas transexuais não podem se relacionar afetivo e sexualmente mutuamente, bem como exercer o direito à parentalidade por meio da gravidez.

Esse momento também foi representado como uma “experiência solitária”, uma vez que, por acreditarem nos riscos de violência transfóbica em espaços públicos pela sua condição de homem grávido, o “isolamento social” torna-se uma realidade durante todo o processo gestacional, podendo estender-se até o puerpério, gerando traumas significativos e repercussões em sua saúde mental. Outro aspecto importante são as relações de apoio junto ao companheiro durante a gestação. Entre os três relatos que estabeleceram relações homoafetivas, entre homens gays (um homem trans e outro cisgênero), dois desses são ancorados por uma “masculinidade tóxica” reproduzida pelo companheiro cisgênero. Essas relações foram sinalizadas pela falta de apoio, e, quando o gestante se sentia apoiado, esse acontecimento relacionava-se à questão “financeira”, entendida como uma “obrigatoriedade” e sendo insuficiente para o homem trans grávido^(3)^.

Percebe-se que as relações homoafetivas e de apoio durante a gestação foram representadas por uma relação “negativa e marcada por conflitos” e que perpassa construções históricas, sociais e culturais sobre os papéis de gênero atribuídos à pessoa gestante. Nesse caso, os homens trans ocupam o papel de “cuidadores”, e seus companheiros cisgêneros reproduzem comportamentos sob o descompromisso com o exercício efetivo da parentalidade ou mesmo desconhecimento e insegurança para reconhecer e assumir seu papel na criação da criança diante do embate com os padrões familiares estabelecidos socialmente.

Destacam-se três relatos em que as relações ocorreram junto aos casais transcentrados, tratando-se de uma configuração em que duas pessoas transexuais e travestis se relacionam afetivo-sexualmente. Foram marcadas pelo “respeito e apoio mútuo” e perpassadas pelo respeito de sua identidade de gênero e da gestação como acontecimento que constituía sua masculinidade, inclusive perpassando as dinâmicas entre familiares. Observa-se, também, que os homens trans representam a gestação enquanto um momento “solitário” e “opressor”. A solidão foi o tema abrangente que permeou as experiências, fazendo com que construíssem estratégias de enfrentamento para gerenciar seus sentimentos. Alguns discursos reforçam que a gravidez foi marcada por uma profunda sensação de “não se reconhecer” e “falta de conexão com um feto em desenvolvimento”, o que vai de encontro a estudo no qual um dos participantes afirmou que “odiava estar grávido” e descreveu uma profunda solidão durante a gravidez^(3,21)^.

### C3) Sentidos e experiências durante a assistência à saúde

Essa categoria relaciona as representações sociais desses sujeitos sobre a gestação ao medo do processo de parturição, que é o momento em que os cuidados de saúde são prestados durante o trabalho de parto e nascimento, sendo ancoradas pela percepção difundida socialmente de que não são capazes biologicamente de realizar um parto normal. Esse fato pode se relacionar à ocorrência de violência obstétrica e transfóbica em instituições marcada por especificidades de gênero, a exemplo das “maternidades”, além da exposição desses corpos “abjetos” e que não são reconhecidos como possíveis de “gestar” entre profissionais de saúde no momento do parto^(8)^.

Observou-se que o conteúdo das representações sociais da assistência à saúde durante a gestação também vinculou-se à preferência do acompanhamento pré-natal em serviço de saúde privado com o médico obstetra e, consequentemente, pela via de parto cesária em detrimento ao parto natural e da assistência obstétrica ofertada pelos serviços do Sistema Único de Saúde (SUS), nesse caso, visto como um local com maior possibilidade de ocorrer violências durante um momento “mágico e desafiador” de sua vida. Estudos mostraram que existem fatores que influenciam a escolha da via de parto, como os aspectos psicoculturais, socioeconômicos, demográficos e, especialmente, interferências médicas, que contribuem, por vezes, para uma decisão direcionada. Por vezes, esses fatores convergem para desnaturalizar o parto vaginal e enaltecer a escolha pelo parto cesáreo; portanto, a decisão pela via de parto nem sempre segue os critérios clínicos de elegibilidade e a vontade inicial da pessoa gestante, comprometendo sua autonomia nesse processo^(22,23)^.

No contexto do cuidado obstétrico, a persistência do modelo de atendimento tecnocrático em serviços de saúde atribui a responsabilidade à instituição e autoridade ao profissional médico sobre a pessoa gestante por meio do “controle” de seus corpos, evidenciada pela passividade de escolha do usuário perante a figura do médico, tida como uma “figura de saber”, em que depositam sua confiança^(24)^. Observa-se a falta de preparo dos parturientes para o trabalho de parto e parto, sendo que a insegurança, a angústia e o medo estão presentes nesse momento. É importante que a equipe de saúde acolha suas demandas, busque conhecer como se deu a assistência durante o pré-natal e trace as estratégias que melhor se adequem a esses indivíduos, considerando suas especificidades. Destaca-se, portanto, que o enfermeiro tem papel fundamental durante esse processo e que vai desde a assistência pré-natal e propaga-se até o puerpério^(25)^.

No nosso estudo, entre os sete homens trans que realizaram consulta pré-natal, apenas um foi acompanhado por enfermeiro que atuava em uma Unidade de Saúde da Família (USF), perpassando por situações de violência transfóbica na assistência pré-natal, como a insistência na solicitação do nome de registro civil para preenchimento de formulários durante o atendimento, não garantindo o respeito ao uso do nome social, quando não retificado, e comprometendo a adesão do gestante ao serviço. Destaca-se a condição de vulnerabilidade socioeconômica que fez com que esse participante procurasse o atendimento no SUS.

Nesse contexto, as representações sociais construídas sobre a violência obstétrica que surgem ao longo dos relatos dos homens trans deste estudo são assimiladas com o não respeito ao nome social durante as consultas pré-natal, desrespeito à identidade de gênero masculina durante o ciclo gravídico-puerperal, deslegitimação da gestação baseada em estereótipos cisgêneros por profissionais da saúde, negação do acesso a serviços de saúde público ou privado destinados apenas a mulheres cisgêneras gestantes, negação do direito do gestante de assinar os documentos disponibilizados pelos espaços de saúde no momento do parto enquanto “pai” e dificuldade em sentar-se em cadeiras preferenciais para gestantes identificadas nos diversos transportes públicos do país.

Apesar disso, foram relatadas experiências positivas durante a assistência à saúde, caracterizadas por encontros clínicos que proporcionaram privacidade, naturalização da gestação trans, reconhecimento de sua parentalidade e ausência de atitudes vexatórias^(26)^. Além disso, a gestação foi assimilada como um acontecimento que possibilitou um “autoconhecimento”, “de mudanças positivas e negativas” e “pedagógica” junto ao reconhecimento da potência do corpo transmasculino em gestar e fazer desse fenômeno um constituinte de sua masculinidade. Esses aspectos têm relação direta com a capacidade que a gestação proporcionou em trazer reflexões importantes para todos que tiveram a oportunidade de compartilhar experiências junto a esse grupo durante a gestação, sejam amigos, familiares ou profissionais de saúde, e que antes não tinham acesso a essas realidades, atuando como um mecanismo de mudança de perspectiva e de transformação social.

### C4) Modificações corporais, emocionais e estratégias de ocultação da gestação

Essa categoria evidencia que as representações sociais da gestação entre os participantes deste estudo se associam às mudanças físicas e emocionais proporcionadas pelo processo gestacional, que por vezes surgem simultaneamente, tendo influência na vivência desses sujeitos. Tais mudanças estão relacionadas a como essas pessoas percebem seu corpo durante a gestação, por meio de um processo de não reconhecimento de sua imagem enquanto homem e reforçando os padrões e papéis de gênero atribuídos à gravidez como “*uma coisa mais feminina que um cara trans pode fazer*” (E1).

Nesse conflito subjetivo, o uso das tecnologias de afirmação de gênero, como o “*binder*”, “a fita micropore”, casacos, blusas de frio e roupas largas, é importante como estratégia de manutenção dos estereótipos construídos sob a lógica da masculinidade hegemônica durante a gestação, ocultando as características físicas proporcionadas pela gravidez e consideradas socialmente como “femininas”, contribuindo para promover uma “passabilidade” desse corpo em espaços públicos, gerando segurança e impactando sua qualidade de vida.

Percebe-se o quanto se torna difícil homens trans lidar com as mudanças corpóreas, principalmente aquelas que se aproximam do simbólico da feminilidade, a exemplo do aumento das mamas, aumento da barriga de “grávido” e ganho ponderal, impactado pela recomendação da interrupção da hormonização masculinizante durante esse período da sua vida e interferindo no processo de afirmação de gênero, através da diminuição de características socialmente vistas como masculinas na materialidade de seus corpos.

Apenas um relato neste estudo diz ter feito uso de hormônios durante a gestação, gerando um “aumento de pelos” na criança após o nascimento. Há escassez de literatura sobre o efeito da afirmação de gênero a partir do uso da testosterona na função reprodutiva de homens trans. Por vezes, profissionais de saúde utilizam dados decorrentes de pesquisas realizadas junto às mulheres cisgêneras, inferindo sobre as recomendações nos cuidados em saúde desse grupo^(27)^. Esses dados corroboram um dos relatos apresentados nesta pesquisa, em que a ideia de fazer uso de testosterona e não menstruar, em decorrência dos efeitos da medicação, não teria riscos de engravidar; no entanto, a testosterona não se trata de anticoncepcional e, apesar de fazer uso para promover características “masculinas”, alguns desses homens mantêm suas funções reprodutivas adequadamente, sendo recomendado o uso de preservativos interno ou externo ou outros métodos contraceptivos^(28)^.

No que diz respeito à amamentação, a realização ou não da cirurgia de mastectomia masculinizadora antes de engravidar foi o principal fator que afetou a decisão em torno dessa experiência. Além disso, o fato de reconhecer a amamentação enquanto uma estratégia para fornecer nutrição teve influências significativas que perpassam sentimentos de “deixar-se de lado” para cumprir com esse papel, que seria “um bem maior” para a criança, podendo essa prática ser ressignificada ao longo do processo gestacional. Neste estudo, dos setes homens trans participantes, cinco deles optaram pela amamentação. Alguns relatos disponíveis na literatura mencionam que muitos se sentem confortáveis em amamentar em espaços públicos, sendo esse um ato político^(29)^.

É preciso utilizar outras possibilidades de alimentação infantil para filhos de homens trans que não optarem pela amamentação, a exemplo do uso dos bancos de leite ou fórmulas lácteas, sendo responsabilidade dos profissionais comunicarem durante a assistência pré-natal a respeito das diferentes opções, não devendo pressionar o usuário e respeitando sua autonomia^(29)^. Nesse contexto, torna-se fundamental o profissional enfermeiro, no exercício de seu papel de cuidador e educador, utilizar a educação em saúde como estratégia para discutir junto ao usuário sobre a importância e as diversas possibilidades de alimentação infantil, considerando sua realidade concreta.

Com o aumento das mamas, percebe-se que esses homens utilizam, frequentemente, estratégias de ocultação desta parte do corpo, por meio do uso do “*binder*” ou “fita micropore”; por vezes, o uso torna-se tão intenso que acaba sendo comparado a uma “tortura”, pelas dores e desconfortos causados. O uso dessas tecnologias resulta em isolamento frequente, devido a um contexto de violências transfóbicas que podem perdurar até o puerpério^(30-33)^. Para alguns homens trans, utilizar essas estratégias de ocultação de características próprias da gestação fizeram parte de sua experiência, a fim de aumentar sentimentos de segurança ao sair em público, sendo o uso de casacos, blusas de frio e roupas largas a principal delas, na tentativa de não promover a visualização das mudanças promovidas pela gravidez. Estudos demonstram que homens trans que passaram por essa experiência notaram que suas gestações eram frequentemente identificadas como obesidade, e geralmente eram mais fáceis de disfarçar com roupas do que a própria mama^(18,29)^.

A partir desse mesmo estudo, aqueles que se passam por homem cisgênero relataram ser percebido como um homem obeso e nunca como uma mulher grávida. Em contrapartida, outros homens trans tornaram-se publicamente visíveis enquanto grávidos, no entanto alguns participantes temeram uma maior probabilidade à violência transfóbica^(26)^. Esse dado corrobora um dos relatos aqui mencionados, em que o fato de ser gestante tem sido algo positivo, gerando sentimentos de “felicidade ao ver a barriga crescer”, não afetando sua masculinidade.

### Limitações do estudo

Este estudo enfrentou desafios devido às limitações associadas ao número restrito de participantes, uma vez que se concentrou em uma população específica e de difícil acesso. Essas dificuldades foram exacerbadas durante o período da pandemia de COVID-19, especialmente devido à vulnerabilidade socioeconômica do contexto. Além disso, ocorreram obstáculos na inclusão de homens transexuais no estudo, pois alguns deles não responderam aos contatos do pesquisador principal ou não manifestaram interesse em participar da pesquisa conduzida por uma pessoa cisgênero.

### Contribuições para a área da saúde, enfermagem ou políticas públicas

Este estudo reforça a importância de uma prática avançada de enfermagem no contexto da assistência pré-natal, obstétrica e puerperal, partindo da perspectiva de uma integralidade no cuidado, da equidade no acesso a serviços e do respeito às diferenças, evidenciando a orientação sexual e a identidade de gênero enquanto Determinantes Sociais da Saúde. Portanto, podem subsidiar futuras reflexões no campo de estudo sobre transmasculinidades, direitos sexuais e reprodutivos com repercussões na formação de políticas públicas em saúde para uma prática equânime, integral, acolhedora e resolutiva à população trans, rompendo estigmas e processos transfóbicos.

## CONSIDERAÇÕES FINAIS

As representações sociais sobre a gestação entre homens trans envolveram um amplo campo de significados, em que foram articulados os esforços deles em “aceitar” a gravidez ao “se deixar de lado” e optar pela continuidade do processo gestacional, sendo representada como uma “experiência solitária”. A assistência à saúde vinculou-se à preferência do acompanhamento pré-natal em serviço privado e, consequentemente, pela via de parto cesária. As mudanças físicas e emocionais proporcionadas pelo processo gestacional foram relacionadas a como essas pessoas percebem seu corpo durante a gestação, por meio de um processo de não reconhecimento de sua imagem enquanto homem e reforçando os padrões e papéis de gênero atribuídos à gravidez como algo feminino.
